# Relevance of diffusion-weighted imaging with background body signal suppression for staging, prognosis, morphology, treatment response, and apparent diffusion coefficient in plasma-cell neoplasms: A single-center, retrospective study

**DOI:** 10.1371/journal.pone.0253025

**Published:** 2021-07-09

**Authors:** Akiko Yamada, Yoichi Araki, Yuko Tanaka, Shunsuke Otsuki, Arisa Yamada, Mitsuru Moriyama, Seiichiro Katagiri, Tamiko Suguro, Michiyo Asano, Seiichiro Yoshizawa, Daigo Akahane, Nahoko Furuya, Hiroaki Fujimoto, Seiichi Okabe, Moritaka Gotoh, Kunihito Suzuki, Kazuhiro Saito, Akihiko Gotoh

**Affiliations:** 1 Department of Hematology, Tokyo Medical University, Tokyo, Japan; 2 Department of Radiology, Tokyo Medical University, Tokyo, Japan; Henry Ford Health System, UNITED STATES

## Abstract

Accurate staging and evaluation of therapeutic effects are important in managing plasma-cell neoplasms. Diffusion-weighted imaging with body signal suppression magnetic resonance imaging (DWIBS-MRI) allows for acquisition of whole-body volumetric data without radiation exposure. This study aimed to investigate the usefulness of DWIBS-MRI in plasma-cell neoplasms. We retrospectively analyzed 29 and 8 Japanese patients with multiple myeloma and monoclonal gammopathy of undetermined significance, respectively, who underwent DWIBS-MRI. We conducted a histogram analysis of apparent diffusion coefficient values. The correlations between each histogram parameter and staging, cell maturation, prognosis, and treatment response were evaluated. We found that the apparent diffusion coefficient values in patients with monoclonal gammopathy of undetermined significance were lower than those in patients with multiple myeloma. Pretreatment apparent diffusion coefficient values of immature myeloma were lower than those of mature myeloma. Moreover, these values decreased in proportion to stage progression in Durie-Salmon classification system but showed no significant correlation with other staging systems or prognosis. Patients were stratified as responder, stable, and non-responder based on the International Myeloma Working Group criteria. The magnitude of changes in apparent diffusion coefficients differed significantly between responders and non-responders (0.154 ± 0.386 ×10–3 mm2/s vs. -0.307 ± 0.424 ×10–3 mm2/s, p = 0.003). Although its usefulness has yet to be established, DWIBS-MRI combined with apparent diffusion coefficient measurement allowed for excellent response evaluation in patients with multiple myeloma. Furthermore, apparent diffusion coefficient analysis using DWIBS-MRI may be useful in predicting cell maturation and total tumor volume.

## Introduction

Magnetic resonance imaging (MRI) is an important method in the diagnosis of hematopoietic tumors, such as malignant lymphoma and multiple myeloma (MM), for both staging and assessing treatment response and recurrence. Diffusion-weighted imaging (DWI) is a technique based on measuring the random Brownian motion of water molecules within a voxel of tissue. Although it was previously used only for imaging the central nervous system, Takahara et al [1] used DWI to image the whole body under spontaneous respiration with background signal suppression. They termed this method diffusion-weighted imaging with background body signal suppression (DWIBS) [1]. DWIBS-MRI has been used to demonstrate a broad range of bone marrow statuses, allowing for lesion extraction and accurate determination of therapeutic effects. Since the publication of this procedure, whole-body MRI (WB-MRI) has become a method of interest in diagnosing a variety of malignant tumors [2]. The usefulness of WB-MRI has been established in MM [3, 4], particularly for the screening of systemic bone lesions at the time of diagnosis [5, 6]. The 2016 edition of the International Myeloma Work Group (IMWG) diagnostic criteria co-listed ^18^F-fluorodeoxyglucose positron emission tomography–computed tomography (FDG-PET/CT) and WB-MRI as the most sensitive tests for detecting bone marrow and extramedullary lesions [7].

The diffusion of water molecules in malignant tumors is suppressed relative to normal tissue. DWI is based on measuring the random Brownian motion of water molecules in interstitial space and tumor tissues. Since the Brownian motion is limited due to high cell density and interstitial pressure, diffusion-weighted images show lesions with restricted diffusion as high-intensity signals with low apparent diffusion coefficient (ADC) values [2]. Recently, it has become easier to assess the ADC value by means of automated applications. In solid tumors that tend to cause bone metastases, such as those in prostate cancer and breast cancer, the usefulness of whole-tumor ADC values for staging and determining treatment response has been reported [8, 9]. In the field of hematology, the usefulness of WB-MRI for assessing response to the treatment of malignant lymphoma with bone marrow invasion has been reported [10]. Prospective studies have shown that ADC is positively correlated with the response to induction therapy in cases of MM [11–16].

In this retrospective study, we intended to review the efficacy of ADC for evaluating treatment response in patients with MM, where its usefulness has been reported. Furthermore, we investigated the usefulness of DWIBS-MRI findings and their correlation with stage, prognosis, and morphology in plasma-cell neoplasms.

## Materials and methods

### Patient cohort and study design

All subjects involved in the study provided their written informed consent about the use of their medical data according to the Declaration of Helsinki. The protocol for this study was approved by the Institutional Review Board of Tokyo Medical University Hospital (approval number: T2019-0198). This study was conducted using anonymized data for analysis.

**Fig 1:**
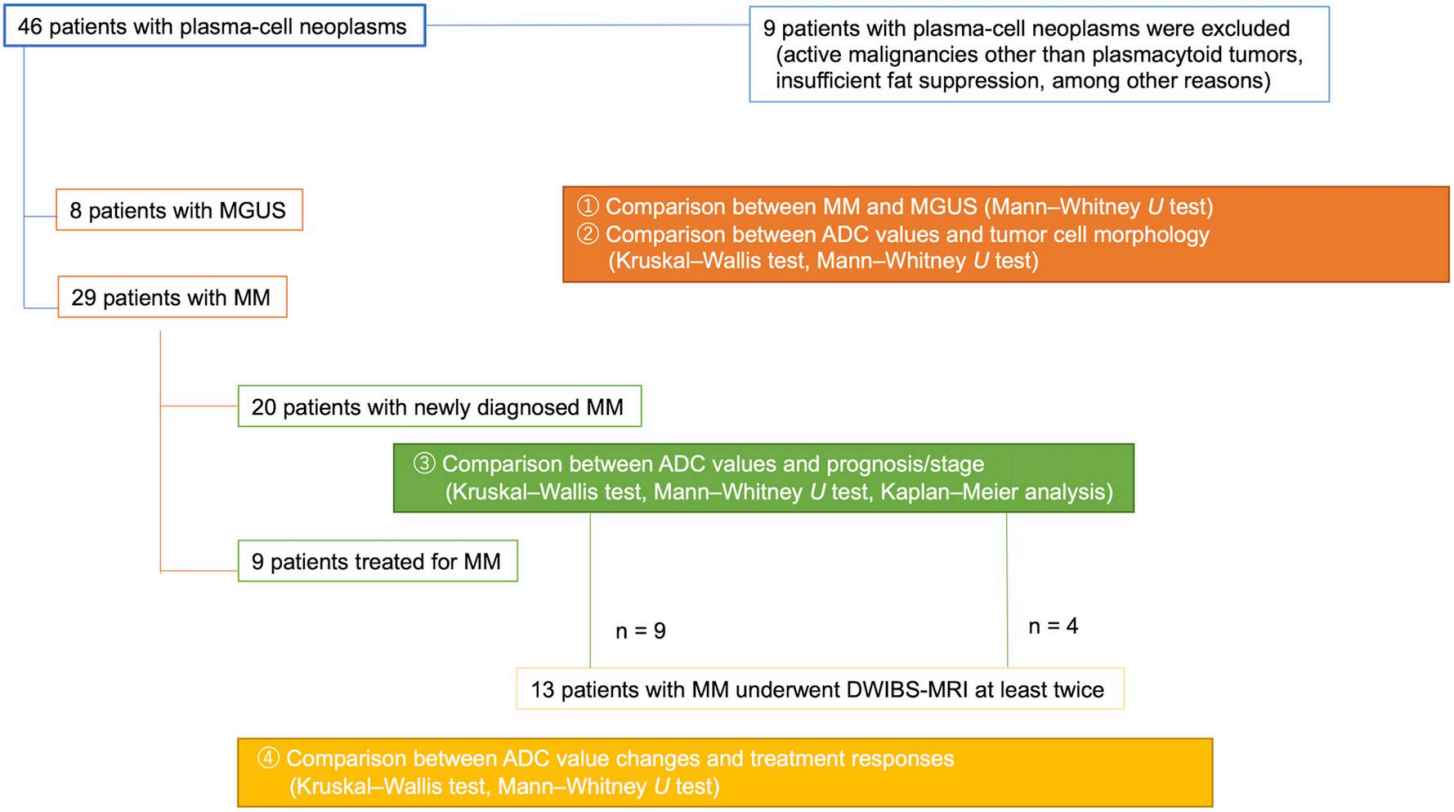
Study flowchart. The object of each study and statistical analysis method are shown. ADC, apparent diffusion coefficient; DWIBS-MRI, diffusion-weighted imaging with body signal suppression magnetic resonance; MGUS, monoclonal gammopathy of undetermined significance; MM, multiple myeloma.

In this retrospective study, 46 Japanese patients with plasma-cell neoplasms, who underwent DWIBS-MRI at our institution in Tokyo between 2017 and 2020 were enrolled. Nine patients with active malignancies other than plasmacytoid tumors at the time of MRI were excluded. Hence, 20 patients with untreated MM (Table 1), 8 with monoclonal gammopathy of undetermined significance (MGUS) (Table 1), and 9 with previously treated MM (Table 2) (Fig 1) were included in this study. Twenty newly diagnosed patients, who underwent DWIBS-MRI at diagnosis were categorized according to the Durie–Salmon (DS) staging system [17], International Staging System (ISS) [18], Revised International Scoring System (R-ISS) [19], and Southwest Oncology Group (SWOG) classifications [20] for analysis (Table 1). We compared the changes in ADC values with the conventional IMWG criteria for treatment response. Overall, 13 patients—including newly diagnosed and previously treated patients—underwent DWIBS-MRI examination 38 times, with each patient undergoing the examination at least twice (Tables 2 and 3). The clinical disease status was determined at each examination visit based on the IMWG criteria. At each DWIBS-MRI exam, patients were categorized into three groups: responder group (partial response [PR] or better, n = 13), stable group (stable disease [SD], n = 12), and non-responder group (progressive disease [PD], n = 13) (Table 3). We compared the change in the absolute ADC value (ΔADC) and ADC percentage (ΔADC%) of each group. In addition, myeloma cells are classified into three subgroups with different degrees of differentiation based on their surface antigens. The immature type had a higher grade of malignancy that affected prognosis [21]. Thus, newly diagnosed MMs were classified into immature, intermediate, and mature types based on the presence or absence of MPC-1 and the expression of adhesion factors CD45 and CD49e [22–24]. The immature type demonstrated a larger nuclear size, narrower cytoplasm, and an increased nucleus-to-cytoplasm (N:C) ratio [25]. Although previous reports on ADC values have mainly evaluated ADC mean values, we used the highest frequency value (ADC_mode_) because, unlike solid tumors, myeloma tumor cells are not homogenous and have varying ADC values.

**Table 1:** Demographic and clinical characteristics of patients with newly diagnosed multiple myeloma and monoclonal gammopathy of undetermined significance.

**Multiple myeloma (N = 20)**	
**Sex, n**	
Male	11
Female	9
**Age, years, mean (range)**	68 (39–88)
**M-protein heavy chain type, n**	
IgG	10
IgA	3
IgD	1
Bence-Jones protein	6
**M-protein light chain type, n**	
κ	13
λ	7
**Durie-Salmon stage, n**	
IA	6
IB	1
IIA	7
IIB	0
IIIA	6
IIIB	0
**International Staging System, n**	
**I**	6
**II**	13
**III**	1
**Revised International Scoring System, n**	
**I**	1
**II**	15
**III**	1
**Unknown**	2
**Southwest Oncology Group staging, n**	
**I**	8
**II**	11
**III**	1
**IV**	0
**Cell morphology, n**	
Immature	9
Intermediate	9
Mature	2
**Type of MRI**	
1.5T	13
3T	7
**Monoclonal gammopathy of undetermined significance (N = 8)**	
**Sex, n**	
Male	6
Female	2
**Age, years, mean (range)**	64 (48–83)
**M-protein heavy chain type, n**	
IgG	7
IgA	1
IgD	0
Bence-Jones protein	0
**M-protein light chain type, n**	
κ	5
λ	2
Unknown	1
**Type of MRI**	
1.5T	4
3T	3

**Table 2:** Treatment responses of patients who underwent diffusion-weighted imaging with body signal suppression magnetic resonance imaging (MRI) repeatedly.

Case number	M-protein type	Sex	Age at diagnosis	Time since diagnosis (days)	Status at first MRI	Post treatment	Time since ASCT (days)
**1**	IgG-κ	M	71	148	PD	RT, Bd	-
**2**	IgA-λ	M	55	115	PD	Bd, Pd, CyBorD	-
**3**	IgA-λ	F	73	1,426	PD	Bd, MD, Rd	-
**4**	IgA-λ	F	64	1,108	Paraprotein relapse	BD, L-PAM, BLd	-
**5**	BJP-κ	M	43	602	PD	BLd, KRd, Pom, KPd, Eld, CPA, DCEP	-
**6**	IgG-κ	M	59	893	PD	Bd, BLd, ASCT	856
**7**	BJP-κ	F	52	2,887	PD	Bd, ASCT, LEN	2,662
**8**	IgG-κ	M	53	304	CR	RT	-
**9**	IgG-λ	F	70	2,083	PD	Bd, VTD, Rd, Pd, PanoBd, KRd, DLd	-
**10**	BJP-κ	F	53	-	Newly diagnosed	-	-
**11**	IgG-λ	F	52	-	Newly diagnosed	-	-
**12**	IgD-κ	M	67	-	Newly diagnosed	-	-
**13**	IgG-κ	F	59	-	Newly diagnosed	-	-

ASCT: autologous stem cell transplantation; BD: bortezomib, high-dose dexamethasone; BLd: bortezomib, lenalidomide, low-dose dexamethasone; Bd: bortezomib, low-dose dexamethasone; CPA: cyclophosphamide; CR; complete response; CyBorD: cyclophosphamide, bortezomib, dexamethasone; DCEP: dexamethasone, cyclophosphamide, etoposide, cisplatin; DLd: daratumumab, lenalidomide, dexamethasone; Eld: elotuzumab, lenalidomide, low-dose dexamethasone; F: female; KPd: carfilzomib, pomalidomide, low-dose dexamethasone; KRd: carfilzomib, lenalidomide, low-dose dexamethasone; L-PAM: melphalan; LEN: lenalidomide; M: male; MD: melphalan, dexamethasone; PD: progressive disease; PanoBd: panobinostat, bortezomib, low-dose dexamethasone; Pd: pomalidomide, dexamethasone; Pom: pomalidomide; RT: radiation therapy; Rd: lenalidomide, low-dose dexamethasone; VTD: bortezomib, thalidomide, dexamethasone.

**Table 3:** Clinical course of patients in terms of treatment response.

Case number		Duration (days)	Treatment regimen	ΔADC_mean_ (×10^−3^ mm^2^/s)	ΔADC_mean%_ (×10^−3^ mm^2^/s)	Group
**1**	1–1	149	DVd*4	-0.00015	-0.01767	Non-responder
**2**	2–1	63	VTD-PACE*2, RT, IT*3	-2.4E-05	-0.00277	Non-responder
**3**	3–1	421	IRd*11	-0.87436	-63.665	Non-responder
	3–2	323	PCd*6, BPd*2	-0.32123	- 0.6437	Non-responder
**4**	4–1	131	BPd*6	-0.00016	-0.00018	Stable
	4–2	55	BPd*2	0.00049	0.000562	Stable
	4–3	153	KRd*5	0.00027	0.000308	Stable
	4–4	267	KRd*9	0.21077	0.243173	Non-responder
	4–5	266	KRd*10	0.2325	0.215777	Stable
**5**	5–1	56	DCEP, DLd*1, ELd+CPA	-0.35904	-41.4475	Non-responder
	5–2	27	MCNU-VMP	0.86600	170.7385	Responder
	5–3	42	1st Allo-SCT by Flu/Mel	-1E-05	-0.00073	Responder
	5–4	96	Daratumumab+RT	-0.73452	-53.5028	Non-responder
	5–5	21	IRd+L-PAM	0.73452	115.0056	Responder
	5–6	28	Dexamethasone	-0.00042	-0.03059	Non-responder
	5–7	27	RT	-0.5068	-36.9178	Non-responder
	5–8	56	2nd Allo-SCT by MEAM	0.5068	58.52329	Responder
	5–9	24	Elo maintenance	-0.86592	-63.08	Non-responder
	5–10	58	RT	0.35988	71.001	Non-responder
**6**	6–1	37	Elo	0.12382	14.28308	Responder
	6–2	298	No treatment	0.123815	14.28308	Stable
	6–3	351	No treatment	-0.11568	-0.11677	Stable
**7**	7–1	439	Bd	-0.05800	-0.10339	Stable
	7–2	77	Pd*3	-0.40295	-0.80109	Responder
	7–3	72	Kd*3	0.12738	1.27315	Stable
**8**	8–1	92	BLd*3	0.50685	58.48657	Responder
	8–2	89	KRd*3	0.00016	0.011649	Responder
	8–3	117	ASCT by HD-LPAM+ KRd maintenance*4	0.11598	8.443322	Responder
	8–4	272	Rd maintenance	-0.11602	-0.07788	Stable
**9**	9–1	161	BLd*7	0.22492	35.05922	Responder
	9–2	394	ASCT by HD-LPAM+ KRd maintenance*6, Pom maintenance	-0.46418	-0.53572	Responder
**10**	10–1	49	Bd*1, VTD-PACE*2	0	0	Responder
	10–2	125	ASCT by HD-LPAM	0	0	Stable
	10–3	38	IRd maintenance*1	-0.6003	-1	Non-responder
**11**	11–1	1,001	Bd*1, BLd*11, PCd*6, BPd*2, PCd*11, Kd*4	-0.0006	-0.0007	Responder
**12**	12–1	708	No treatment	0.13191	0.260177	Stable
	12–2	168	No treatment	0.22709	0.355433	Stable
**13**	13–1	280	Kd*12	-0.508	-1	Non-responder

ASCT: autologous stem cell transplantation, Allo-SCT: allogenic stem cell transplantation; BLd: bortezomib, lenalidomide, low-dose dexamethasone; BPd: bortezomib, pomalidomide, low-dose dexamethasone; Bd: bortezomib, low-dose dexamethasone; CPA: cyclophosphamide; DCEP: dexamethasone, cyclophosphamide, etoposide, cisplatin; DLd: daratumumab, lenalidomide, dexamethasone; DVd: daratumumab, bortezomib, low-dose dexamethasone; ELd; elotuzumab, lenalidomide, low-dose dexamethasone; Elo: elotuzumab; Flu: fludarabine; HD-LPAM: high-dose melphalan; IRd: ixazomib, lenalidomide, low-dose dexamethasone; IT: intrathecal chemotherapy; KRd: carfilzomib, lenalidomide, low-dose dexamethasone; Kd: carfilzomib, dexamethasone; L-PAM: melphalan; MCNU-VMP: ranimustine, vincristine, melphalan, prednisolone; MEAM: ranimustine, etoposide, cytarabine, melphalan; Mel: melphalan; PCd: pomalidomide, cyclophosphamide, dexamethasone; Pd: pomalidomide, dexamethasone; Pom: pomalidomide; RT: radiation therapy; Rd: lenalidomide, dexamethasone; VTD-PACE: bortezomib, thalidomide, dexamethasone, cyclophosphamide, etoposide, cisplatin, doxorubicin.

### DWIBS-MRI technique

Whole-body MRI was performed on the 3- and 1.5-Tesla systems (Vida, Avanto fit; Siemens Medical Solutions, Erlangen, Germany). Each parameter is shown in Table 4. DWIBS data were processed using BD Score (Pix Space Ltd., Fukuoka, Japan).

**Table 4:** Imaging protocols for the DWIBS experiments.

	Avanto 1.5T	Vida 3T
Sequence type	Short T1 version recovery-echo planar imaging	Short T1 version recovery-echo planar imaging
phase encoding direction	anteroposterior	anteroposterior
coil	32ch spine coil and 18ch body matrix coil×2	32ch spine coil and 18ch body matrix coil×2
field of view	500mm, 60slices × 4steps	430 mm, 30 slices ×7 steps
matrix	128 × 128	150×120
time (TR/TE)	7470/58 ms	4830/67 ms
inversion time	180 ms	230 ms
slice thickness	5 mm	5 mm
bandwidth/pixel	3,256Hz	2,084 Hz
flip angle	90°	90°
EPI factor	80	120
total imaging time	7 min 28 s	8 min 20 s
b value	0 and 800 mm^2^/s	0 and 800 mm^2^/s

Whole body image was drawn on the DWIBS (b value = 0, 800 mm^2^/s) image; next, ADC was automatically converted to the ADC map [26]. The ADC region of 0.4–1.5 mm^2^/s was extracted and analyzed. Thus, ADC_maximum_, ADC_minimum_, ADC_average_, ADC_mean_, ADC_mode_, kurtosis, skewness, and total diffusion volume (tDV, in mL) were analyzed and compared. In addition, during the follow-up of each patient, the same equipment used during the previous visit was used.

### Statistical analyses

Statistical analysis was performed using IBM SPSS version 26 (IBM, Armonk, NY, USA). Kruskal–Wallis tests and Mann–Whitney *U* tests were used to compare ADC values, tDV, serum free-light chain (FLC), and staging grade. Kaplan–Meier curves were used to analyze overall and progression-free survival. Statistical significance was defined as p < 0.05.

## Results

### Comparison of ADC values between MM and MGUS

**Fig 2:**
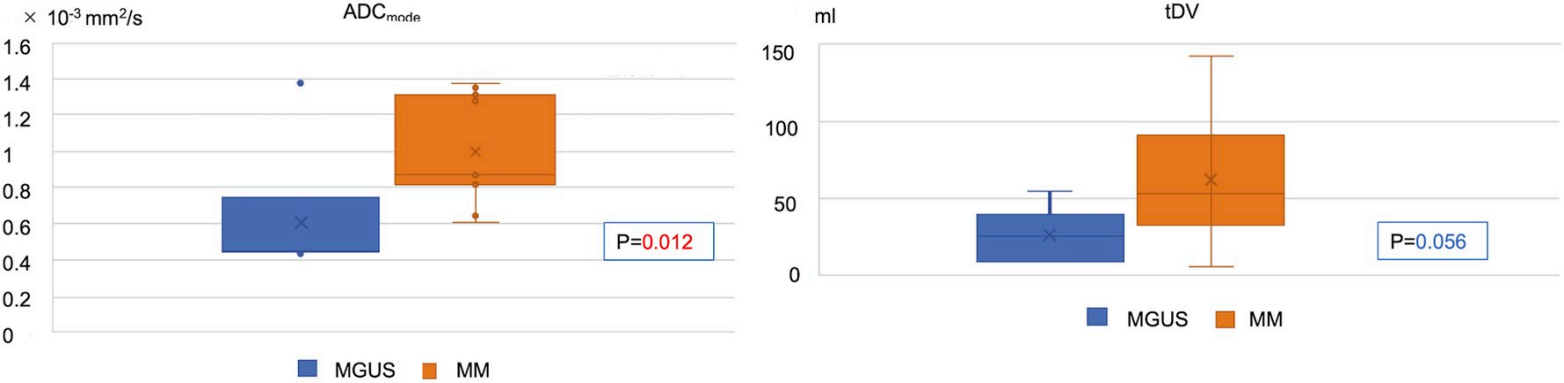
Stages of plasmacytoid tumor and apparent diffusion coefficient (ADC) value. The ADC_mode_ was significantly higher in patients with MM than in those with MGUS (1.00 ± 0.28 ×10^−3^ mm^2^/s vs. 0.61 ± 0.38 ×10^−3^ mm^2^/s). tDV tended to be higher in patients with MM than in those with MGUS (62.3 ± 40.3 ml vs 25.9 ± 18.1 ml). MM, multiple myeloma; MGUS, monoclonal gammopathy of undetermined significance; tDV, total diffusion volume.

This study included 20 newly diagnosed patients with MM and 8 who were untreated for MGUS (Fig 1; Table 1). There was no significant difference in age or sex between these groups. In patients with MM, ADC_mode_ at the time of diagnosis was significantly higher than that in patients with MGUS. The tDV tended to be higher in patients with MM than in those with MGUS (Fig 2). In solid tumors, the ADC level tended to decrease with increase in tumor grade [27, 28]; however, this was not observed in MM and MGUS.

### Myeloma cell morphology and ADC

**Fig 3:**
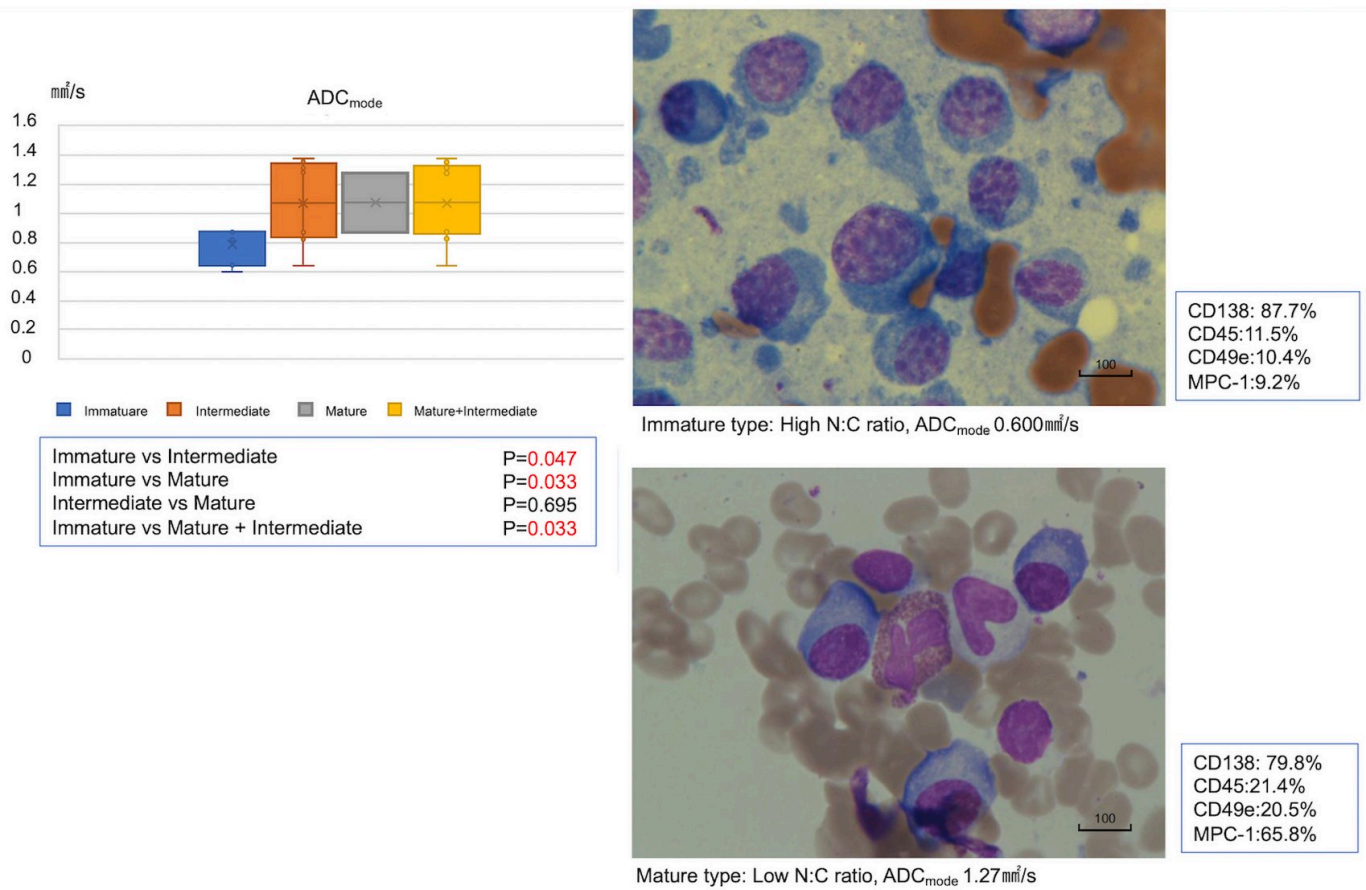
Myeloma cell morphology and apparent diffusion coefficient (ADC) values. The ADC_mode_ was 1.10 ± 0.26 ×10^−3^ mm^2^/s in the mature myeloma cell type, 0.99 ± 0.40 ×10^−3^ mm^2^/s in the intermediate myeloma cell type, and 0.82 ± 0.17 ×10^−3^ mm^2^/s in the immature myeloma cell type. ADC values of the intermediate and mature cell types were significantly higher than those of the immature cell type. N:C ratio, nucleus-to-cytoplasm ratio.

Based on bone marrow aspiration performed at the same time as DWIBS-MRI, myeloma cell samples from 20 patients with newly diagnosed MM were classified and their ADC values were examined. The ADC values of intermediate and mature cell types were significantly higher than those of the immature cell type (Fig 3). No association between cell morphology and prognosis was found (overall survival and progression-free survival analysis using the Kaplan–Meier method; S1 Fig).

### Correlation between ADC values and prognosis or clinical staging in newly diagnosed MM

**Fig 4:**
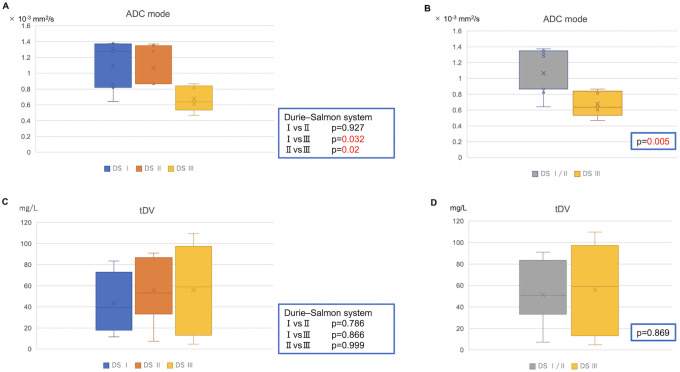
Durie–Salmon classification and apparent diffusion coefficient (ADC) values. **(A)** The ADC_mode_ was 1.10 ± 0.12 ×10^−3^ mm^2^/s in stage I, 1.04 ± 0.09 ×10^−3^ mm^2^/s in stage II, and 0.68 ± 0.27 ×10^−3^ mm^2^/s in stage III. **(B)** The ADC_mode_ was 1.08 ± 0.27 ×10^−3^ mm^2^/s in stages I and II. ADC values of Durie–Salmon stage I and II were significantly higher than those of stage III. **(C)** and **(D)** tDV tended to be higher in Durie–Salmon stage III than in stages I and II but no significant differences were observed (stage I: 43.4 ± 29.8 ml, stage II: 55.9 ± 29.6 ml, stages I and II: 49.6 ± 29.8 ml, stage III: 56.0 ± 19.4 ml). DS, Durie–Salmon; tDV, total diffusion volume.

The associations between each classification and the ADC value were examined. ADC values decreased proportionally to stage progression according to the Durie–Salmon classification but showed no significant correlation with other staging systems (Fig 4).

### Evaluation of treatment effectiveness and ADC

**Fig 5:**
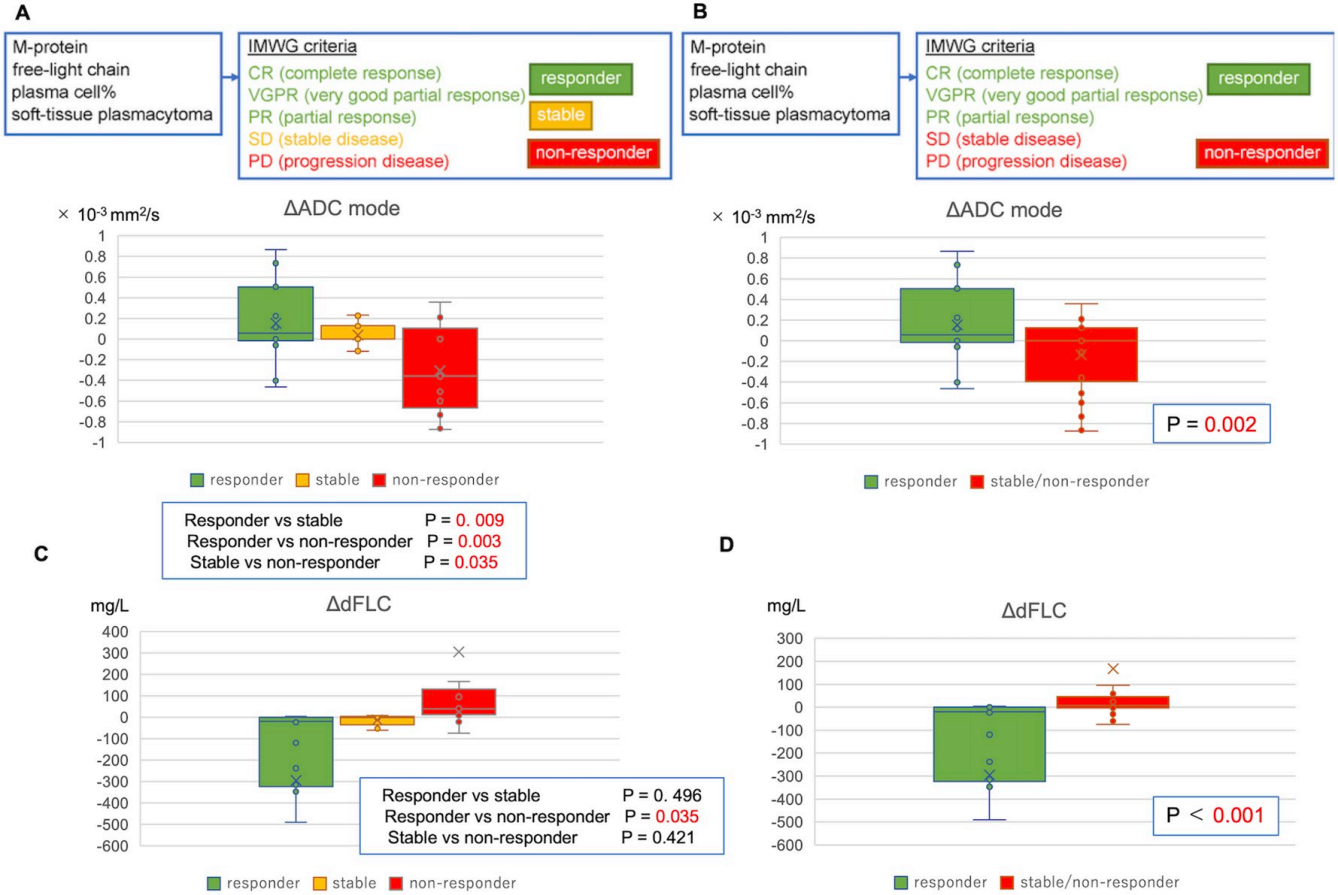
Treatment effectiveness and apparent diffusion coefficient (ADC) values. **(A)** Comparison between the responder (partial response or better, N = 13, ADC_mode_: 0.154 ± 0.386 ×10^−3^ mm^2^/s), stable (stable disease, N = 12, ADC_mode_: 0.038 ± 0.111 ×10^−3^ mm^2^/s), and non-responder (progressive disease, N = 13, ADC_mode_: -0.307 ± 0.424 ×10^−3^ mm^2^/s) groups. **(B)** Comparison between the responder (N = 13, ADC_mode_: 0.154 ± 0.386 ×10^−3^ mm^2^/s) and non-responder + stable (N = 25, ADC_mode_: -0.135 ± 0.351 ×10^−3^ mm^2^/s) groups. ΔADC_mode_ was significantly higher in the responder group than that in the stable + non-responder group. **(C)** Average dFLC in the responder (-294.98 ± 677 mg/L), stable (-13.11± 25.11 mg/L) and non-responder (305.87 ± 715.10 mg/L) groups increased with a worsening in disease prognosis, albeit no significant difference was observed. **(D)** dFLC differed significantly between the responder (-294.98 ± 677 mg/L) and non-responder + stable (167.18 ± 552 mg/L) groups. dFLC, difference free-light chain; IMWG, International Myeloma Work Group.

Among the three groups, responder, stable, and non-responder, the ΔADC _mode_ and ΔADC _mode%_ values in the responder group were significantly higher than those in the non-responder group (Fig 5A). Furthermore, we compared responders with a combined stable + non-responder group and found a clear stratification (Fig 5B). The difference in FLC (dFLC)—the earliest indicator of treatment response in MM—and ΔADC differed significantly between the responder and stable + non-responder groups (Fig 5C and 5D, S2 Fig); however, there was no correlation between the two indicators (spearman’s rho correlations: r_2_> -0.015, p> 0.1). Hence, both ΔADC and dFLC were useful independent predictors of response. For example, in case 5 (Table 3), new plasmacytomas and pancytopenia occurred despite the absence of a serum FLC change after the second allo-stem cell transplantation. However, ADC was decreased. There may be situations in which ΔADC is more useful than dFLC, such as in the evaluation of cases of clonal changes and in the transition to hyposecretory or non-secretory forms during treatment.

## Discussion and conclusions

MRI is more widely available and costs 80%–85% less than PET-CT. Additionally, MRI is a short duration exam that involves no pre-exam dietary restrictions or radiation exposure. These reduced financial and physical burdens on the patient are advantageous. In this study, we experienced a case in which a lesion was false-positive on PET-CT and true-negative on MRI. Although PET-CT has been considered superior to MRI for the early determination of post-treatment efficacy [29], the usefulness of DWIBS-MRI early in the treatment period deserves to be directly studied based on future case accumulation. To date, it remains unclear which modality is more useful. In two previous retrospective studies comparing the ability of WB-MRI and PET-CT to assess bone infiltration in MM patients, WB-MRI showed higher sensitivity than did PET-CT in detecting both diffuse infiltration and focal lesions [30, 31].

Although no universal reference ADC values exist because the ADC is determined by the reference value set by the scanner model, several previous reports using *in vivo* data have shown no significant difference in ADC values between 1.5T and 3T for normal tissues and malignancies [32–35]. Hence, in this study we performed the analysis using both 1.5T and 3T ADC values. Moreover, benign lesions with inflammatory tissue can also have low ADC values due to the suppression of water diffusion, and lead to false-positive results [36]. Further studies are warranted to clarify the evaluation of ADC.

The main objective of this study was to investigate the usefulness of DWIBS-MRI and ADC value in evaluating treatment efficacy for MM. Zhang et al. [15] performed a pre- and post-treatment ADC comparison after using bortezomib-based induction. They defined patients with a very good partial response (VGPR) or better as “deep responders” and those with a PR or worse as “non-deep responders.” In contrast to our findings, they observed that post-treatment ADC_mean_ remained unchanged in responders but increased in non-deep responders [10]. Differences in case-grouping criteria and inclusion of patients with PR in the “non-deep responders” group may explain this disparity. We observed no significant differences between patients with PD and those with SD in our study; thus, the comparison between the responder and stable + non-responder groups clarified the stratification. In patients with MM, the early achievement of VGPR or better improves prognosis, and the comparison between deep and non-deep responders is an important target for remission induction. Further, ADC analysis may be more useful for deciding whether to change treatment. A clear-cut deep responder is easy to diagnose based on serum FLC and immunofixation, as well as with the introduction of the concept of minimal residual disease (MRD), without considering ADC values [37]. Nonetheless, deciding whether it is necessary to increase treatment intensity in older patients who are not eligible for stem cell transplantation and are at risk of adverse events remains an important aspect of MM treatment, and ADC analysis may facilitate clinical decision-making for patients with stable or unpredictable diseases. The value of MRI as an imaging biomarker for response assessment in MM patients has been reviewed recently [38]. In our study, FLC-centered evaluation was negative in one stable case, and this parameter did not reach statistical significance. Future studies should investigate whether ΔADC reflects the clinical course of patients with SD.

In our study, we observed significant differences in ADC changes between the responder and non-responder groups, indicating that DWIBS-MRI examination combined with ADC measurement allowed for an excellent short-term treatment response evaluation in patients with MM. However, unlike previous studies [11–16], this study was retrospective, had a limited number of patients owing to the single-center study design, with inconsistent timing of examination. MRI timing was determined at the time of the initial diagnosis based on one of the following scenarios: (i) when the doctor deemed the current treatment ineffective, (ii) when the data indicated SD or PD, or (iii) when only a short time had elapsed since treatment change. These inconsistencies may have affected the statistical power of our findings. Nonetheless, considering that in real-life clinical situations DWIBS-MRI may be performed as needed instead of at predetermined times, this imaging modality may be useful for when a hematologist requires disease evaluation, such as when considering a change in treatment regimen or during ongoing treatment follow-up, as was observed in this study.

**Fig 6:**
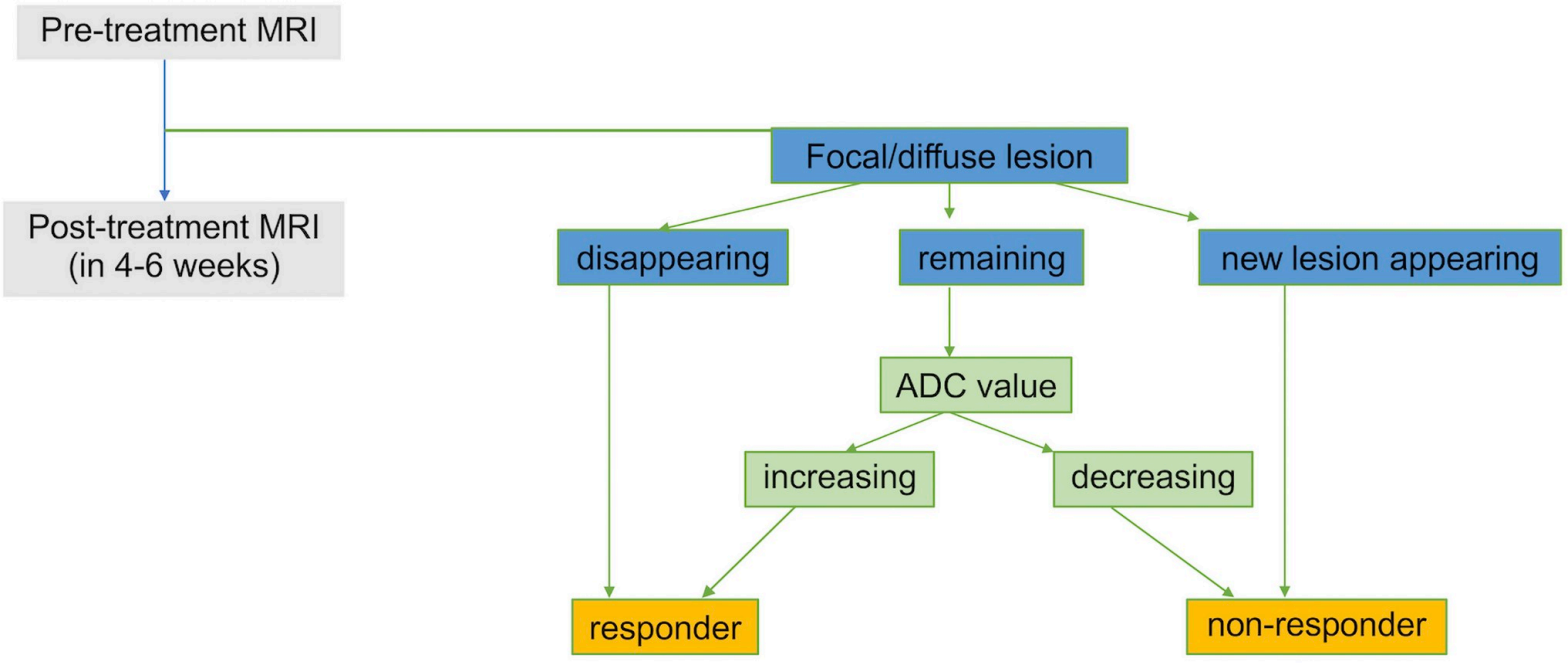
Proposed flowchart for treatment response assessment using DWIBS-MRI. This proposed flowchart evaluates treatment effectiveness using MRI findings and ADC values. Patients considered “non-responders” should be candidates for treatment change. ADC, apparent diffusion coefficient; DWIBS, diffusion-weighted imaging with body signal suppression; MRI, magnetic resonance imaging.

The results obtained from this study are limited by a small number of cases. Despite an insufficient number of cases for statistical analysis, we believe that our findings indicate that DWIBS-MRI may be as useful as previously reported. In this study, we observed several cases in which ADC changes preceded changes in M-protein and FLC, even when the interval between examinations was as short as 30 days, suggesting that DWIBS-MRI can effectively predict early response to treatment. Furthermore, an increase in ADC relatively soon after treatment is a highly reliable indicator, and further ADC reduction in non-responders may reflect disease progression. This should be evaluated comprehensively by considering FLC and other factors as well as MRI findings. Thus, we propose a flowchart for evaluating treatment effectiveness based on MRI findings (Fig 6).

To the best of our knowledge, no study has described a correlation between myeloma cell morphology and ADC values. In our study, we observed that immature cells had low ADC values. The proliferation of tumor cells causes an increase in intracellular structures and a narrowing of the stroma, limiting the movement of water molecules. Thus, differences in proliferative capacity, tumor cell size, and N:C ratio affect the ADC [39, 40]. Furthermore, we analyzed the overall and progression-free survival of 86 MM patients, who were diagnosed from 2009 to 2019 in our hospital and classified in groups according to cell morphology and staging. As reported for solid tumors [27, 28], our results suggest a correlation between differentiation and grade of myeloma and ADC (Fig 3); however, we found that the patient population diagnosed and treated at our facility was not stratified by conventional prognostic indicators (Durie–Salmon classification and overall survival are shown in S3 Fig). In particular, the overall survival of patients in a group that was previously considered to have a poor prognosis was prolonged compared with that of those classified according to the original Durie–Salmon system. We hypothesized that recent drug developments may have contributed to this improvement in prognosis. Future studies should determine the validity of these prognostic factors.

To the best of our knowledge, no published study has included an ADC analysis for patients with MGUS, to date. A study observed that healthy adults tended to have lower ADC_mean_ values than MM patients (0.73 ± 0.05 ×10^−3^ mm^2^/s vs. 0.86 ± 0.12 ×10^−3^ mm^2^/s, p = 0.061) [16] while comparing the ADC values of healthy adults and MM patients. Normal bone marrow is low in water content due to its high fat cell content, resulting in a low ADC. We hypothesized that, as MM progresses, the percentage of adipocytes in the bone marrow decreases by the replacement of MM cells, resulting in a relative increase in water content and consequently, an increase in ADC values. Although our study did not include healthy adults and the comparison between ADC values of patients with MGUS and healthy adults is a subject for future studies, we found that patients with MGUS had lower ADC values than those with MM. This result suggests that MGUS lesions have more similarities to normal bone marrow than MM lesions. Furthermore, ADC_mode_ values differed significantly according to Durie–Salmon staging (Durie–Salmon stage I was considered early phase MM) and tDV tended to increase with Durie–Salmon stage prognosis, albeit without significant differences. Durie–Salmon stage reflects tumor volume. These findings suggest that low ADC values indicate an increase in tumor volume or density.

**Fig 7:**
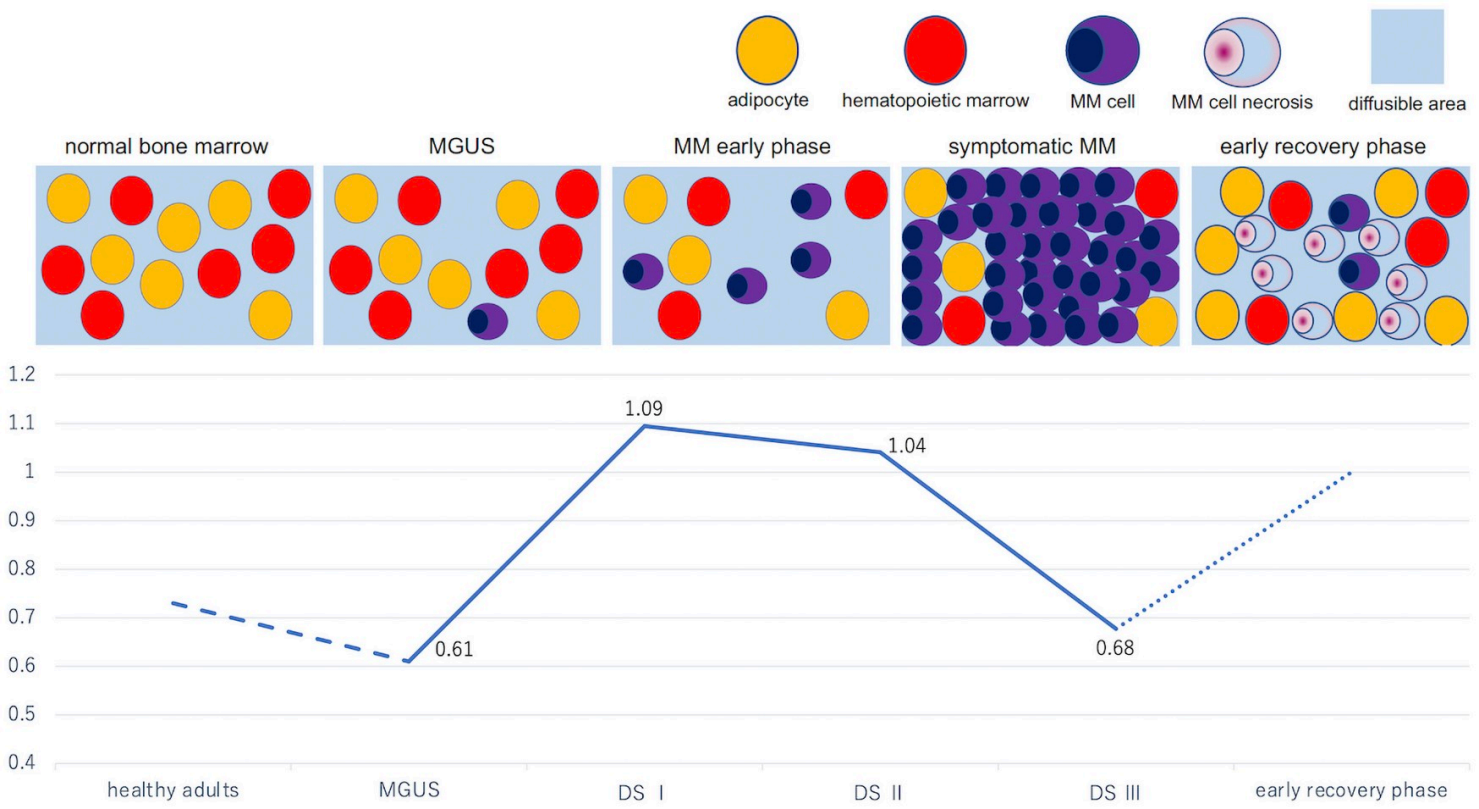
Proposed changes in bone marrow appearance and apparent diffusion coefficient (ADC) values according to pathology. Normal bone marrow is low in water content due to its high fat cell content, resulting in a low ADC. In MGUS or early phase MM, the percentage of adipocytes in the bone marrow decreases, resulting in a relative increase in water content and, consequently, an increase in ADC values. In symptomatic MM, diffusion limitation owing to abnormal proliferation of neoplastic plasma cells in the bone marrow may result in a decline in ADC values. This diagram includes ADC values reported by Giles et al. [16] and in our study. ADC values for healthy adults were reported as average instead of mode, hindering a direct comparison; thus, we used dotted lines to differentiate these values. DS, Durie–Salmon; MGUS, monoclonal gammopathy of undetermined significance; MM, multiple myeloma.

As the disease progresses, diffusion limitation due to abnormal proliferation of neoplastic plasma cells in the bone marrow may result in a decline in ADC. Several reports, including the Myeloma Response Assessment and Diagnosis System (MY-RADS), have reported that, in the early phase (4–6 weeks) after treatment, abnormal accumulations in the bone marrow due to tumor cell death, hemorrhage, edema, and recovery of the adipocyte percentage after tumor cell loss may be involved in ADC increase [41]. Furthermore, ADC may subsequently decrease during the process of recovery to normal bone marrow because of increasing adipocytes [42]. Fig 7 presents a schematic diagram of changes in ADC values in plasma-cell neoplasms, which are worth considering based on previous reports and the results of our study.

In the current era of new drug development, the prognosis of patients with MM is improving. However, even if the bone marrow responds to treatment, it may still develop disease progression, such as the transition to non-secretory or hyposecretory MM or the appearance of plasmacytoma. In such cases, laboratory findings, including FLC and bone marrow puncture, are limited in their ability to evaluate clonal changes in myeloma cells with disease progression. In terms of a minimally invasive, less time-consuming, and less costly approach, DWIBS-MRI performed at the appropriate time could provide a more accurate assessment of treatment efficacy, allowing for a more appropriate selection of treatment and a better prognosis.

## Supporting information

S1 Fig Overall survival and cell morphology. There was no stratification in cell morphology.
(TIF)

S2 Fig Evaluation using apparent diffusion coefficient (ADC)_mean_. ΔADC_mean_ for responder, 0.103 ± 0.191 ×10^−3^ mm^2^/s; for non-responder + stable, -0.117 ± 0.209 ×10^−3^ mm^2^/s. ΔADC_average_ for responder, 0.144 ± 0.235 ×10^−3^ mm^2^/s; for non-responder + stable, -0.126 ± 0.270 ×10^−3^ mm^2^/s.
(TIF)

S3 Fig Overall survival and Durie–Salmon staging system. Conventional prognostic indicators were analyzed for the target group of this study and all cases in our facility between 2009–2020. No classification presented a clear stratification. As an example, the Durie–Salmon classification system is shown. MM, multiple myeloma.
(TIF)
