# Supplementary figures and images for: Relevance of diffusion-weighted imaging with background body signal suppression for staging, prognosis, morphology, treatment response, and apparent diffusion coefficient in plasma-cell neoplasms: A single-center, retrospective study
Source: PLoS One. 2021 Jul 9;16(7):e0253025. doi: 10.1371/journal.pone.0253025 (PMC8270139; doi:10.1371/journal.pone.0253025)

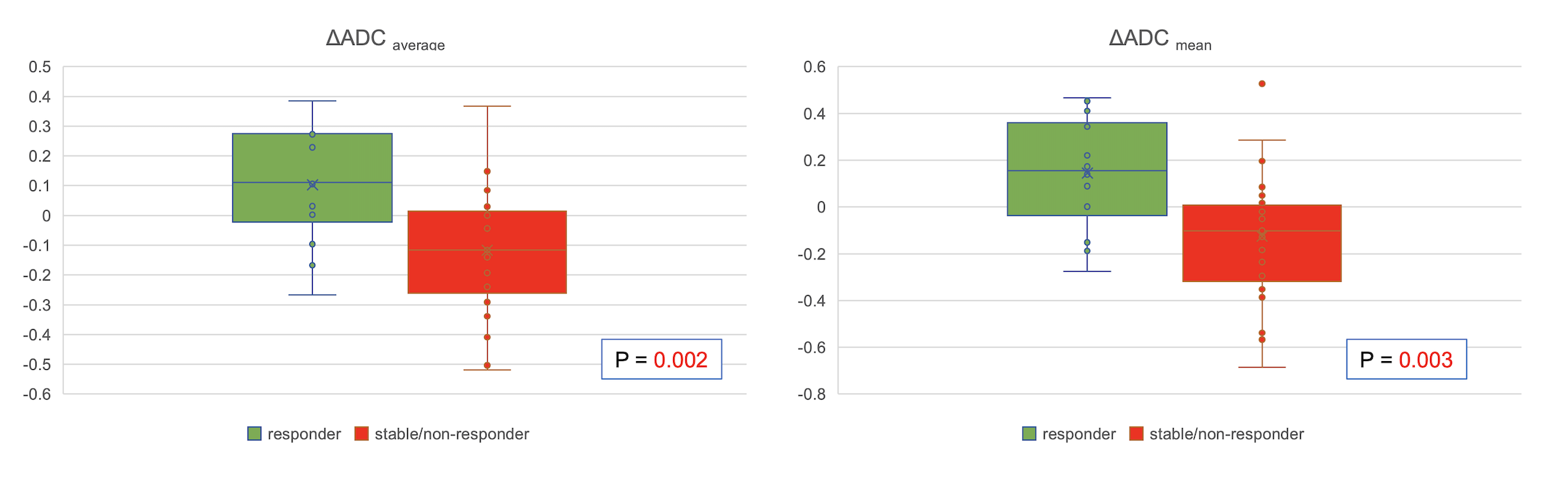

Supplement: S1 Fig — There was no stratification in cell morphology. (TIF) [file pone.0253025.s001.tif]

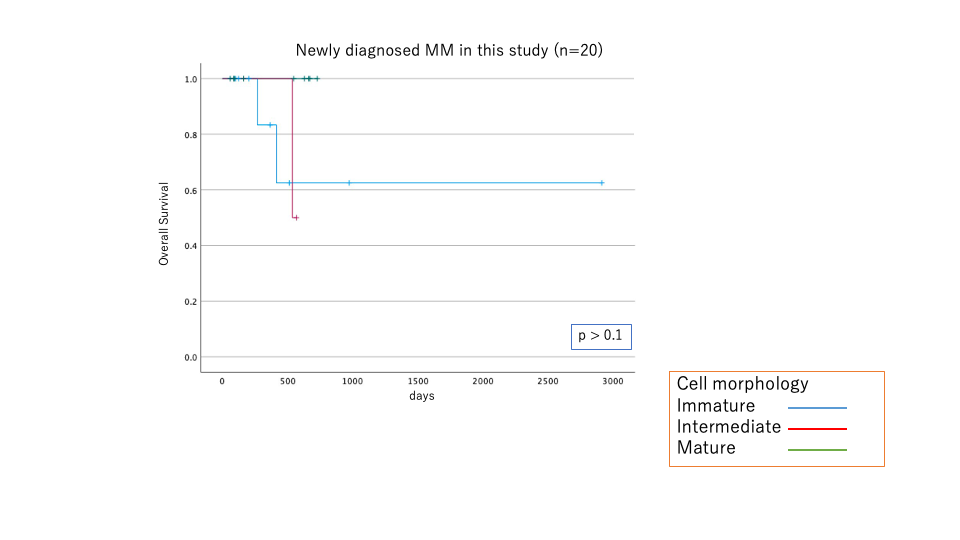

Supplement: S2 Fig — ΔADCmean for responder, 0.103 ± 0.191 ×10^−3^ mm2/s; for non-responder + stable, -0.117 ± 0.209 ×10^−3^ mm2/s. ΔADCaverage for responder, 0.144 ± 0.235 ×10^−3^ mm2/s; for non-responder + stable, -0.126 ± 0.270 ×10^−3^ mm2/s. (TIF) [file pone.0253025.s002.tif]

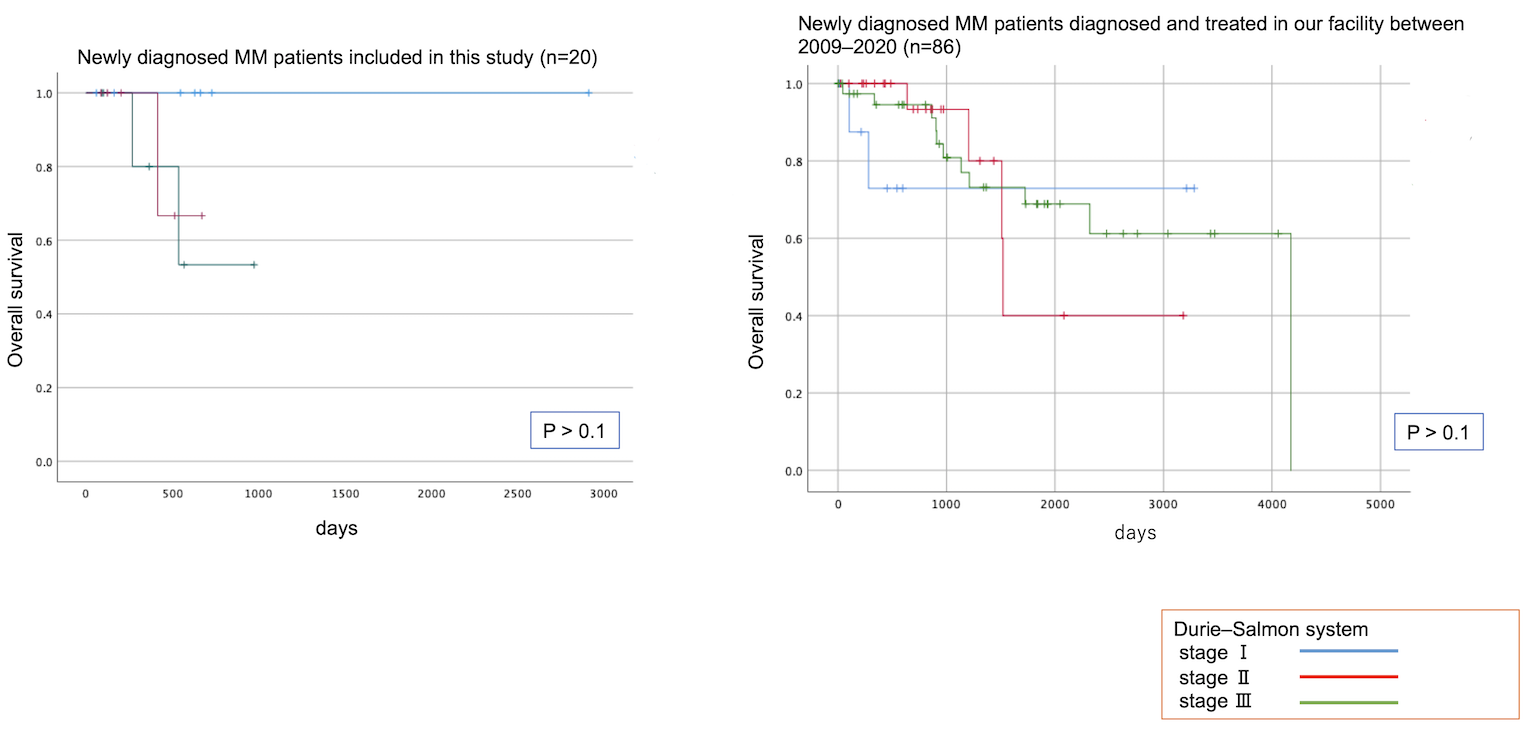

Supplement: S3 Fig — Conventional prognostic indicators were analyzed for the target group of this study and all cases in our facility between 2009–2020. No classification presented a clear stratification. As an example, the Durie–Salmon classification system is shown. MM, multiple myeloma. (TIF) [file pone.0253025.s003.tif]
